# The Hypolipidemic Activity of Boronated Nucleosides in Male Mice and Rats

**DOI:** 10.1155/MBD.1996.173

**Published:** 1996

**Authors:** Bruce S. Burnham, Anup Sood, Jeno Tomasz, W. J. Powell, Bernard F. Spielvogel, S. Y. Chen, Iris H. Hall

**Affiliations:** 1 Division of Medicinal Chemistry and Natural Products School of Pharrnacy University of North Carolina Chapel Hill North Carolina 27599-7360 USA; 2 Boron Biologicals Inc. 620 Hutton St. Raleigh NC 27606 USA

## Abstract

The boronated nucleosides with varying bases and sugar moieties were shown to be potent hypolipidemic agents in rodents. The 3′– aminocynaoborane dideoxythymidine derivative caused reductions in serum cholesterol and triglyceride levels, tissue lipids, VLDL and LDL cholesterol levels while elevating HDL cholesterol levels in rodents. The agents suppressed rat hepatic acetyl CoA synthetase, HMG-CoA reductase, acyl-CoA cholesterol acyl transferase, phosphatidylate phosphohydrolase and lipoprotein lipase activities while elevating cholesterol-7α-hydroxylase activity from 25 to 100 μM.


### THE HYPOLIPIDEMIC ACTIVITY OF BORONATED NUCLEOSIDES IN MALE MICE AND RATS

Bruce S. Burnham[1], Anup Sood[2], Jeno Tomasz[2], W. J. Powell[2], Bernard F. Spielvogel[2], S. Y. Chen[1] and Iris H. Hall[1]*

[1] Division of Medicinal Chemistry and Natural Products, School of Pharmacy University of North Carolina, Chapel Hill, North Carolina 27599-7360, USA
[2] Boron Biologicals, Inc., 620 Hutton St., Raleigh, NC 27606, USA

**KEY WORDS:** hypolipidemia, boronated nucleoside, 3'-aminocyanoborane dideoxythymidine

### Abstract:
The boronated nucleosides with varying bases and sugar moieties were shown to be potent hypolipidemic agents in rodents. The 3'-aminocynaoborane dideoxythymidine derivative caused reductions in serum cholesterol and triglyceride levels, tissue lipids, VLDL and LDL cholesterol levels while elevating HDL cholesterol levels in rodents. The agents suppressed rat hepatic acetyl CoA synthetase, HMG-CoA reductase, acyl-CoA cholesterol acyl transferase, phosphatidylate phosphohydrolase and lipoprotein lipase activities while elevating cholesterol-7α-hydroxylase activity from 25 to 100 μM.

### Introduction:

Previously 2'-deoxyribonucleoside cyanoboranes and phosphate-boronated nucleotides were shown to be hypolipidemic agents in rodents at 8 mg/kg/day ip and orally [1]. In rats the cholesterol levels of VLDL and LDL fractions were reduced and the HDL-cholesterol content was significantly increased with reductions of both serum cholesterol and triglyceride levels after 14 days at 8 mg/kg/day of these boronated deoxyribonucleosides. These boronated nucleosides and nucleotides were shown to be safe for therapeutic use [2]. We have now expanded the types of nucleosides with boron substitutions at different nitrogen moieties. The current investigation is an effort to establish their hypolipidemic activity in mice and rats at 8 mg/kg/day.

### Methods:

Source of materials:
All of the compounds have previously been synthesized and the chemical and physical characteristics reported [3-5]. All isotopes were purchased from New England Nuclear. Substrates and co-factors were obtained from Sigma Chemical Co. Sprague Dawley male rats were obtained from Charles River Laboratory. $CF_1$ mice were obtained from Jackson Laboratory. Animals were maintained in light cycles of 12 h at 22°C. Rats were maintained in individual wire cages and mice were housed three/plastic cage. Food (Agway/Prolab Animal Diet) and water were *ad libitum.*

Normalipidemic studies:
For structure activity studies, $CF_1$ male mice (~28g) were administered boronated nucleosides in 1% CMC at 8 mg/kg/day, ip. Blood samples were obtained on days 9 and 16 between 7:30 and 8:30 a.m. Daily dosing of the agents was between 9:00 and 10:00 a.m. The serum was obtained by centrifuging the blood for 10 min. at 3500 g. The serum cholesterol levels were determined by a modification of the procedure Liebermann-Burchard reaction [6]. Serum triglyceride levels were determined using a commercial kit [Boehringer Mannheim Diagnostics]. Sprague Dawley male rats (~230 g) were administered orally Compound 6 at 8 mg/kg/day, for two weeks. Weekly blood samples were obtained by tail vein bleeding.

Animal weight, organ weight and food consumption effects:
Control and treated normalipidemic Sprague Dawley male rat(~230g) weights were obtained and expressed as a percentage of the initial body weight (week zero). Food consumption (gm/day/rat) was noted for two weeks for control and treated rats[1].

Tissue lipid levels:
Normalipidemic Sprague Dawley male rats (~230 g) which were treated orally for two weeks with compound 6 at 8 mg/kg/day, were sacrificed and tissue samples of the liver, small intestinal mucosa and aorta were removed. A 24 hr fecal sample was also obtained. A 10% homogenate in 0.25 M sucrose + 0.001 M EDTA, pH 7.2, was prepared for each tissue. An aliquot (2 ml) of the homogenate was extracted [7-8] and the number of mg of lipid extracted was weighed. The lipid residue was taken up in methylene chloride and the levels of cholesterol [6], triglycerides , neutral lipids[9] and phospholipids[10]were determined. Protein content of the whole homogenate was determined [11].

Serum lipoprotein fractions:
Normalipidemic Sprague Dawley male rats(~230g) treated for two weeks with compounds 6 at 8 mg/kg/day, orally were anesthetized with ether and blood (~10 ml) was collected from the abdominal vein. Serum was separated from whole blood by centrifugation at 3500 rpm. Aliquots of the serum were separated into chylomicrons, VLDL, HDL and LDL by ultracentrifugation as modified for normal rats [12, 13]. Each of the fractions was analyzed for cholesterol, triglyceride, neutral lipids, phospholipid and protein levels.

Enzymatic studies:
Compounds 3, 6, 13 and 14 were examined for their ability to inhibit *in vitro* activities of hepatic enzymes involved in lipid synthesis and metabolism. *In vitro* enzymatic studies were performed using 10% homogenates of liver from normalipidemic Sprague Dawley male rats (~230g). The liver homogenates were prepared in 0.25 M sucrose + 0.001 M (ethylenedinitrilo)tetraacetic acid [EDTA], pH 7.2. Acetyl coenzyme A synthetase [14] and adenosine triphosphate dependent citrate lyase activities [15] were determined spectrophotometrically at 540 nm as the hydroxylamate of acetyl coenzyme A formed after 20 min. at 37°C. Cholesterol-7a-hydroxylase activity was determined using [1,2-$^3$H]cholesterol (60 mCi/mmol) [20], and acyl CoA cholesterol acyl transferase [ACAT] activity was determined using [1-$^{14}$C]oleic acid (56.7 mCi/mmol)[16]. Cholesterol synthesis was measured using [1-$^{14}$C] acetyl CoA (62 mCi/mmol) and a post-mitochondrial supernatant (9000 g x 20 min) which was incubated for 60 min. at 37°C [17]. The digitonide derivative of cholesterol was isolated and counted [18]. Cholesterol ester hydrolase activity was determined using 1-$^{14}$C cholesterol oleate [56.6 mCi/mol] [19].

For acetyl carboxylase activity, the enzyme had to be polymerized for 30 min. at 37°C and then the assay mixture containing sodium $^{14}$C-bicarbonate (41.0 mCi/mmol) was added and incubated for 30 min. at 37°C with test drugs[20]. *sn*-Glycerol-3-phosphate acyl transferase activity was determined with *sn*-glycerol-3-phosphate [L-2-3H(N)] (7.1 Ci/mmol) with the microsomal fraction of liver homogenates. The reaction was terminated after 60 min and the lipids were extracted with chloroform/methanol (2:1) containing 1% HCl and counted[21]. Phosphatidylate phosphohydrolase activity was measured as inorganic phosphate released over 60 min. [22]. The released inorganic phosphate after color development with ascorbic acid and ammonium molybate was quantitated at 820 nm. Hepatic lipoprotein lipase was determined using glycerol-tri-$^{14}$C-palmitate [64 mCi/mol] emulsified with lecithin by the method of Chait et al.[23]. Protein content of the liver homogenates was determined [11].

Data denoted in Tables 1-5 represent the means ± standard deviations of each group expressed as a percentage of the control value. The Student's "t" test was applied between control groups and the individual drug treatment groups using the raw data.

Results

The boronated nucleosides demonstrated potent hypolipidemic activity in mice at 8 mg/kg/day ip. Compounds **1**, **3**, **4**, **5**, **6**, **11**, **12**, **13**, **14**, and **15** decreased serum cholesterol levels at least 40% by day 16 [Table 1]. Serum triglyceride levels were reduced greater than 30% on day 16 by compounds **3**, **7**, **12**, **13**, **14**, and **15**. All of the derivatives afforded better hypolipidemic activity than the standards clofibrate at 150 mg/kg/day or lovastatin at 8 mg/kg/day in mice.

Compound 6 was selected for further studies as being representative of this class of derivatives. This compound reduced serum cholesterol levels in rat 36% and serum triglyceride levels were reduced 32% on day 14 after oral administration [Table 2].

Rat tissue lipids after 14 days of drug administration were not elevated. Aorta wall cholesterol was reduced 24% after administration of compound 6 [Table 3]. Fecal triglycerides and phospholipids were elevated significantly after 14 days, i.e. 104% and 50%, respectively while cholesterol levels were reduced 24%. Small intestinal mucosa and aorta wall triglyceride levels were reduced as was aorta wall phospholipids and protein content.

Figure 1: Structures of Boronated Nucleosidyl & Nucleotidyl Hypolipidemic Agents

| Compound # | Structure | Compound # | Structure |
|---|---|---|---|
| 1 |  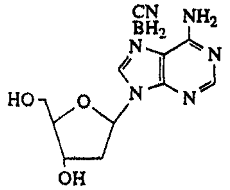 | 2 |  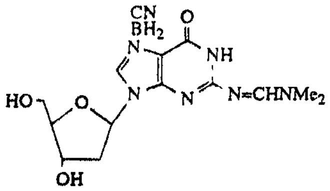 |
| 3 |  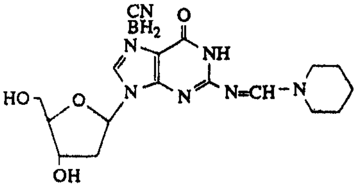 | 4 |  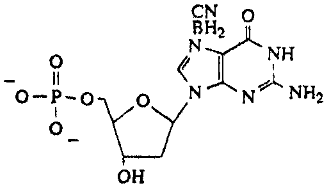 |
| 5 |  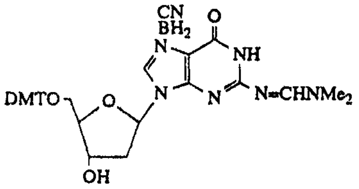 | 6 |  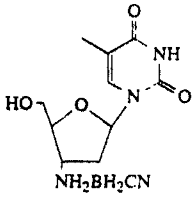 |
| 7 |  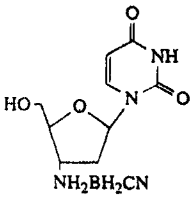 | 8 |  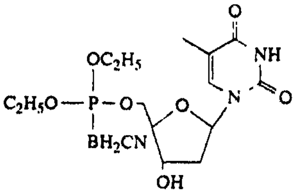 |
| 9 |  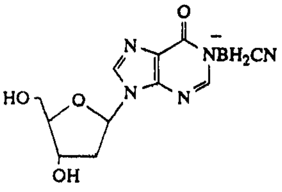 | 10 |  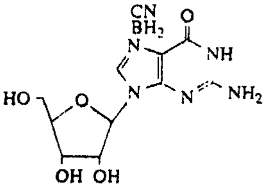 |
| 11 |  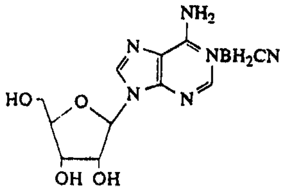 | 12 |  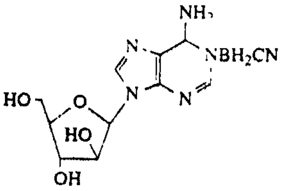 |

Figure 1: Structures of Boronated Nucleosidyl & Nucleotidyl Hypolipidemic Agents (contd.)

| Compound # | Structure | Compound # | Structure |
| --- | --- | --- | --- |
| 13 |  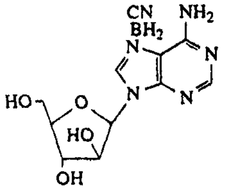 | 14 |  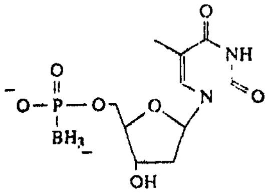 |
| 15 |  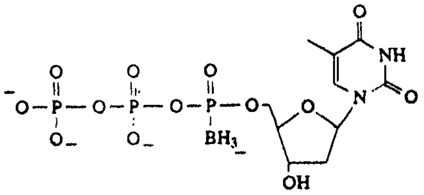 | | |

**Table 1:** *In Vivo* Hypolipidemic Activity of Cyanoborane Adducts in $CF_1$ Male Mice at 8 mg/kg/day, ip for 16 Days.

| *N*=6 Compound # | Percent of Control (*X*±*S.D.*) Day 9 Serum Cholesterol | Day 16 Serum Cholesterol | Day 16 Serum Triglyceride |
|---|---|---|---|
| 1 | 79±5 | 57±4* | 72±4* |
| 2 | 87±6 | 70±4* | 83±5 |
| 3 | 81±4 | 60±3* | 47±3* |
| 4 | 59±5* | 54±3* | 81±5 |
| 5 | 78±4* | 55±4* | 73±3* |
| 6 | 94±5 | 65±3* | 70±3* |
| 7 | 78±4* | 75±5* | 64±6* |
| 8 | 84±5 | 75±4* | 87±5 |
| 9 | 75±5* | 72±4* | 78±4* |
| 10 | 73±4* | 73±3* | 74±3* |
| 11 | 76±4* | 60±4* | 70±4* |
| 12 | 55±3* | 46±3* | 66±3* |
| 13 | 49±4* | 48±3* | 64±5* |
| 14 | 54±4* | 41±3* | 60±4* |
| 15 | 54±6* | 51±3* | 56±3* |
| Control[a] | 100±5[d] | 100±7[e] | 100±6[f] |
| Lovastatin[b] | 85±4 | 82±5* | 86±7 |
| Clofibrate[c] | 87±6 | 78±6* | 75±6* |

[a] 1% CMC; [b] Dosed at 8 mg/kg/day; [c] Dosed at 150 mg/kg/day; [d] 125 mg/dL serum cholesterol; [e] 128 mg/dL serum cholesterol; [f] 137 mg/dL serum triglyceride; *$p \leq 0.001$, Student's "*t*" test

**Table 2:** *In Vivo* Hypolipidemic Activity of the Cyanoborane Adduct of 3'-Amino Dideoxythymidine(6) in Sprague-Dawley Male Rats at 8mg/kg/day Orally for 14 Days.

| *N*=6 Compound | Food Consumption[a] | Percent of Control (*X*±*S.D.*) Day 7 Serum Cholesterol | Day 7 Serum Triglyceride | Day 14 Serum Cholesterol | Day 14 Serum Triglyceride |
|---|---|---|---|---|---|
| Compound 6 | 100+7 | 87±4 | 86±7 | 64±9* | 68±6* |
| Control[b] | 100±6 | 100±6[f] | 100±4[g] | 100±6[h] | 100±5[i] |
| Lovastatin[c] | --- | 85±4 | 91±5 | 82±5 | 86±7 |
| Gemfibrozil[d] | 91±5 | 91±5 | ---- | 82±7 | 62±5* |
| Clofibrate[e] | --- | 89±7 | 83±6 | 86±5 | 74±7* |

[a] Control = 22.1±1.3 g/day/rat; [b] 1% CMC; [c] Dosed at 8 mg/kg/day; [d] Dosed at 90 mg/kg/day; [e] Dosed at 150 mg/kg/day; [f] 73 mg/dL total serum cholesterol [g] 75 mg/dL total serum cholesterol; [h] 111 mg/dL serum triglyceride [i] 112 mg/dL serum triglyceride; *$p \leq 0.001$, Student's "*t*" test

**Table 3:** *In Vivo* Effects of the Cyanoborane Adduct of 3'-Amino Dideoxythymidine on Tissue Lipids in Sprague-Dawley Male Rats After 14 Days at 8 mg/kg/day, Orally

*N*=8 Percent of Control ($X \pm S.D.$)

| | Mg Lipid Extracted | Cholesterol | Triglycerides | Neutral Lipids | Phospholipids | Protein |
|---|---|---|---|---|---|---|
| **Liver** | | | | | | |
| Control | 100 ± 4[a] | 100 ± 5[b] | 100 ± 7[c] | 100 ± 6[d] | 100 ± 7[e] | 100 ± 6[f] |
| Cmp'd **6** | 96 ± 6 | 104 ± 6 | 110 ± 4 | 95 ± 7 | 99 ±6 | 89 ± 6 |
| **Small Intestine** | | | | | | |
| Control | 100 ± 8[g] | 100 ± 7[h] | 100 ± 6[I] | 100 ± 6[j] | 100 ± 6[k] | 100 ± 7[l] |
| Cmp'd **6** | 86 ± 5 | 112 ± 6 | 81 ± 7 | 99 ± 7 | 100 ± 5 | 98 ± 6 |
| **Aorta** | | | | | | |
| Control | 100 ± 4[m] | 100 ± 5[n] | 100 ± 6[o] | 100 ± 5[p] | 100 ± 6[q] | 100 ± 6[r] |
| Cmp'd **6** | 94 ± 7 | 76 ± 5* | 75 ± 4* | 99 ± 7 | 87 ± 7 | 84 ± 7 |
| **Feces** | | | | | | |
| Control | 100 ± 8[s] | 100 ± 6[t] | 100 ± 6[u] | 100 ± 8[v] | 100 ± 8[w] | 100 ±6[x] |
| Cmp'd **6** | 94 ± 8 | 76 ± 6* | 204 ± 10* | 98 ± 7 | 150 ± 6* | 98 ± 5 |

concentration/ gram wet tissue:

| | |
|---|---|
| [a] 50.5 mg lipid | [m] 67.5 mg lipid |
| [b] 9.18 mg cholesterol | [n] 5.77 mg cholesterol |
| [c] 6.37 mg triglyceride | [o] 9.85 mg triglyceride |
| [d] 15.70 mg neutral lipid | [p] 15.28 mg neutral lipid |
| [e] 27.19 mg phospholipid | [q] 28.8 mg phospholipid |
| [f] 12.02 mg/protein | [r] 11.71 mg/protein |
| [g] 68.20 mg lipid | [s] 11.58 mg lipid |
| [h] 12.02 mg cholesterol | [t] 2.84 mg cholesterol |
| [i] 11.20 mg triglyceride | [u] 1.86 mg triglyceride |
| [j] 16.98 mg neutral lipid | [v] 3.39 mg neutral lipid |
| [k] 20.06 mg phospholipid | [w] 5.70 mg phospholipid |
| [l] 42.0 mg protein | [x] 6.99 mg/protein |

*$p \leq 0.001$, Student's "t" test

Rat serum lipoproteins after 14 days administration of compound **6** showed a 24%, 20% and 12% reduction in cholesterol levels of chylomicrons, VLDL and LDL fractions while HDL-cholesterol content was elevated 65% [Table 4]. Triglyceride content was increased in the chylomicron but lowered in the VLDL and HDL fractions. Phospholipids were lower in the chylomicron and VLDL fractions but was elevated in the LDL fraction. Protein content was reduced in the chylomicron and VLDL fractions but was elevated in the LDL and HDL fractions.

*In vitro* hepatic enzyme activities showed similar patterns of effects for the four compounds tested over 60 min [Table 5]. Cytoplasmic acetyl CoA synthetase activity was suppressed significantly by all of the agents following a concentration dependent response. ATP-dependent citrate lyase activity was also reduced by the compounds except by compound **14**. HMG-CoA reductase activity was suppressed moderately by 19% by compound 3 at 100 μM but compounds **13** and **14** caused greater than 50% reduction. Acyl-CoA cholesterol acyl transferase activity was reduced 32-37% by compounds **6** and **14** and 47% by compounds **3** and **13** at 100 μM. Cholesterol ester hydrolase activity was suppressed 37% by compound **6** and 62% by compound **3**. Cholesterol-7α-hydroxylase activity was elevated 12-60% by the agents at 100 μM.
Acetyl-CoA carboxylase activity was elevated by compounds **3** and **13** at all concentrations whereas compounds **6** and **14** caused only 15% reduction at 100 μM. *sn*-Glycerol-3-phosphate acyl transferase activity was essentially unaffected by the compounds. Phosphatidylate phosphohydrolase activity was markedly reduced by compounds **3**, **13** and **14** from 72% to 85% whereas compound **6** caused only 18% reduction. Hepatic lipoprotein lipase activity was reduced 47-48% by compounds **3** and **13** and 24% by compound **6** but the activity was elevated 127% by compound **14** at 100 μM.

### Discussion

The boronated nucleosides were potent hypolipidemic agents in rodents at 8 mg/kg/day both by po and ip administration. The $N^7$ boronated guanosine derivative 3 as well as 3'-aminocyanoborane dideoxythymidine **6**, the

**Table 4:** In Vivo Effects of Cyanoborane Adduct of 3'-Amino Dideoxythymidine **6** on the Lipid Content of Serum Lipoproteins in Sprague Dawley Male Rats After 14 Days at 8mg/kg/day, Orally

*N*=8 Percent of Control ($X \pm S.D.$)

| | Cholesterol | Triglycerides | Neutral Lipids | Phospholipids | Protein |
|---|---|---|---|---|---|
| **Chylomicron** | | | | | |
| Control | $100 \pm 6^a$ | $100 \pm 7^b$ | $100 \pm 7^c$ | $100 \pm 6^d$ | $100 \pm 5^e$ |
| Compound 6 | $76 \pm 5^*$ | $138 \pm 8^*$ | $82 \pm 6$ | $55 \pm 4^*$ | $79 \pm 6$ |
| **VLDL** | | | | | |
| Control | $100 \pm 4^f$ | $100 \pm 5^g$ | $100 \pm 7^h$ | $100 \pm 7^i$ | $100 \pm 5^j$ |
| Compound 6 | $80 \pm 4^*$ | $86 \pm 6$ | $101 \pm 6$ | $60 \pm 5^*$ | $54 \pm 5^*$ |
| **LDL** | | | | | |
| Control | $100 \pm 5^k$ | $100 \pm 6^l$ | $100 \pm 6^m$ | $100 \pm 6^n$ | $100 \pm 8^o$ |
| Compound 6 | $88 \pm 4$ | $99 \pm 7$ | $94 \pm 6$ | $158 \pm 8^*$ | $132 \pm 6^*$ |
| **HDL** | | | | | |
| Control | $100 \pm 6^p$ | $100 \pm 7^q$ | $100 \pm 6^r$ | $100 \pm 7^s$ | $100 \pm 6^t$ |
| Compound 6 | $165 \pm 8^*$ | $68 \pm 5^*$ | $96 \pm 5$ | $103 \pm 6$ | $164 \pm 7^*$ |

per ml serum a 337 μg cholesterol b 420 μg triglyceride c 67 μg neutral lipid d 149 μg phospholipid e 184 μg protein f 190 μg cholesterol g 22 μg triglyceride h 98 μg neutral lipid i 26 μg phospholipid j 50 μg protein

* $p \leq 0.001$, Student's "*t*" test k 210 μg cholesterol l 45 μg triglyceride m m 10 μg neutral lipid n 41 μg phospholipid o 122 μg protein p 544 μg cholesterol q 630 μg triglyceride r 27 μg neutral lipid s 153 μg phospholipid t 657 μg protein

**Table 5:** *In Vitro* Effects on Liver Enzyme Activities of Hypolipidemic Nucleoside -cyanoboranes

*N*=6

| | | Percent of Control ( X + S.D.) | | | | | |
|---|---|---|---|---|---|---|---|
| | | Compound 3 | | | Compound 6 | | |
| Enzyme | Control | 25μM | 50μM | 100μM | 25μM | 50μM | 100μM |
| Acetyl-CoA Synthetase | 100±7[a] | 63±4* | 23±2* | 7±1* | 89±6 | 81±6 | 78±5* |
| ATP-dependent -Citrate Lyase | 100±5[b] | 84±5* | 76±5* | 52±4* | 96±4 | 91±6 | 72±5* |
| HMG CoA Reductase | 100±7[c] | 139±5* | 136±4* | 81±4* | 99±6 | 102±6 | 108±5* |
| Acyl CoA Cholesterol- -acyl Transferase | 100±6[d] | 87±5 | 77±6* | 53±4* | 86±5 | 106±6 | 68±7* |
| NeutralCholesterol Ester Hydrolyase | 100±5[e] | 93±5 | 46±4* | 38±3* | 73±6* | 64±5* | 63±6* |
| Cholesterol-7-alpha-hydroxylase | 100±6[f] | 96±4 | 112±4 | 112±5 | 107±6 | 128±6* | 132±5* |
| Acyl CoA Carboxylase | 100±5[g] | 396±9* | 283±8* | 253±7* | 88±6 | 88±4 | 85±5 |
| *sn*-Glycero-1,3- Phosphate Acyl Transfease | 100±6[h] | 127±6* | 119±5 | 107±6 | 113+7 | 108±6 | 104±5 |
| Phosphatidylate Phosphohydrolase | 100±5[j] | 46±4* | 28±4* | 17±2* | 107±6 | 86±5 | 82±4* |
| Lipoprotein Lipase | 100±6[k] | 66±5* | 54±4* | 53±3* | 99±7 | 83±6 | 74±6* |

[a] 28.5 mg acetyl CoA formed/g wet tissue; [b] 30.5 mg citrate hydrolyzed/g wet tissue; [c] 384900 dpm cholesterol formed/g wet tissue; [d] 224000 dpm/mg of microsomal protein [e] 56436 dpm/mg wet tissue; [f] 4808 dpm/mg of microsomal protein; [g] 537800 dpm/mg wet tissue; [h] 302010 dpm/mg wet tissue;[i] 16.7 μg $P_i$ released/g wet tissue; [j] 278538 dpm/g wet tissue. * $p \leq 0.001$, Student's "*t*" test

*N*=6

| | | Percent of Control (*X*±*S.D.*) | | | | | |
|---|---|---|---|---|---|---|---|
| | | Compound 13 | | | Compound 14 | | |
| Enzyme | Control | 25μM | 50μM | 100μM | 25μM | 50μM | 100μM |
| Acetyl CoA Synthetase | 100±7[a] | 49±4* | 30±2* | 2±1* | 80±5 | 43±3* | 36±3* |
| ATP-dependent Citrate Lyase | 100±5[b] | 67±5* | 58±5* | 55±3* | 117±6 | 96±5 | 88±4 |
| HMG CoA Reductase | 100±7[c] | 59±5* | 56±4* | 45±2 | 58±4* | 52±4* | 49±4* |
| Acyl CoA Cholesterol Acyl Transferase | 100±6[d] | 87±6 | 68±6* | 53±3* | 78±5* | 63±4* | 63±3* |
| Neutral Cholesterol Ester Hydrolase | 100±5[e] | 139±8 | 91±6 | 89±7 | 141±14* | 98±5 | 87±5 |
| Cholesterol-7-alpha- Hydroxylase | 100±6[f] | 111±7 | 160±8* | 160±7* | 96±5 | 100±6 | 122±6 |
| Acyl CoA Carboxylase | 100±5[g] | 357±9* | 299±9* | 168±8* | 115±6 | 100±4 | 85±5 |
| *sn*-Glycero-1-3-Phosphate Acyl Transferase | 100±6[h] | 104±5 | 92±6 | 86±4 | 94±5 | 92±5 | 88±5 |
| Phosphatidylate Phosphohydrolase | 100±5[j] | 46±4* | 24±3* | 15±2* | 78±5* | 35±4* | 28±3* |
| Lipoprotein Lipase | 100±6[k] | 181±7 | 49±4* | 52±4* | 150±8* | 190±8* | 227±9* |

boronated adenosine arabinoside **13** and thymidine 5'-boranophosphate **14** demonstrated good activity in lowering both serum cholesterol and triglyceride levels after 16 days in mice. The boronated thymidine mono and triphosphates were very effective agents but the ribose-nucleosides were not as potent hypolipidemic agents at this dose. Enzymatic studies demonstrated that four of these derivatives inhibited rat hepatic cytoplasmic acetyl-CoA synthetase markedly and ATP-dependent citrate lyase activity moderately which would reduce acetyl-CoA necessary for both fatty acid and cholesterol syntheses. The reduction of HMG-CoA reductase activity, the regulatory enzyme for cholesterol synthesis, by some of the agents should add to the overall reduction of tissue, lipoprotein and serum cholesterol levels. The agents, **13** and **14**, which demonstrated good inhibition of the activity of this enzyme were more effective in lowering cholesterol levels than the compounds, **3** and **6**, which only lowered acetyl CoA synthetase activity. All of the agents lowered hepatic acyl-CoA cholesterol acyl transferase activity, the enzyme responsible for cholesterol ester synthesis. Assuming that the same inhibition occurred in the aorta wall, of this enzyme activity by the agents then plaque growth should be reduced since their size depends directly on the deposition of cholesterol esters. Cholesterol-7-α-hydroxylase activity was elevated by the agents. This is the regulatory enzyme for converting cholesterol to bile acids for excretion into the bile. The lowering of triglyceride levels by the agents is probably due to their ability to reduce the activity of phosphatidylate phosphohydrolase, the enzyme responsible for conversion of phospholipids to triglycerides. The reduction of hepatic lipoprotein lipase by three of the agents would lower the removal of triglycerides from lipoproteins for delivery to the liver or other tissues.

*In vivo* studies with the 3'-aminocyanoborane- dideoxythymidine showed that rat serum cholesterol and triglyceride levels were reduced at 8 mg/kg/day and was comparable in hypolipidemic effects to lovastatin at 8 mg/kg/day and clofibrate at 150 mg/kg/day in rats.

This agent, compound **6**, achieved lowering of VLDL and LDL cholesterol content while elevating HDL cholesterol levels. Modifying this ratio is important in choosing a hypolipidemic therapeutic agent in that VLDL and LDL lipoprotein conduct cholesterol to peripheral tissues including the aorta walls leading to deposition and tissue accumulation whereas HDL conducts free cholesterol from peripheral tissue to the liver for excretion in the bile. Hyperlipidemic patients have high VLDL and LDL cholesterol content and low HDL-cholesterol content; thus, if a therapeutic agent reverses this ratio this is useful clinically [24]. This compound effectively lowered aorta wall cholesterol levels after 14 days administration but there was no observable elevation in cholesterol excretion in the feces on day 14, but triglyceride excretion and phospholipid excretion was elevated at this time. The pharmacological properties of these boronated nucleosides as hypolipidemic agents are similar to those of boronated derivatives reported previously [1]. These studies demonstrate that the compound have potential as hypolipidemic agents but further structure activity relationship studies are need before an agent can be selected for clinical development.

### References

1.Hall I.H., Burnham B.S., Rajendran K.G., Chen S.Y., Sood A., Spielvogel B.F., Shaw B.R. (1993) Biomed. Pharmacother. **47**,79.

2.Hall I.H., Burnham B.S., Chang J.J., Sood A., Spielvogel B.F (1994) Metal-Based Drugs 1, 19.

3.Hall I.H., Hall E.S., Chi L.K., Sood A., Spielvogel B.F. (1992) Anticancer Res. **12**, 1091.

4.Sood A., Spielvogel B.F., Shaw B.R., Carlton L.D., Burham B.S., Hall E.S., Hall I.H. (1992) Anticancer Res. **12**, 335.

5.Sood A., Spielvogel B.F., Shaw B.R., Hall E.S., Chi L.K., Hall I.H. (1992) der Pharm **47**, 833.

6.Ness A.T., Pastewka J.V., Peacock A.C. (1959) Clin. Chem.Acta. **10**, 229.

7.Bligh E.G., Dyer W.J. (1959) J. Biochem. Physiol. **37**, 911.

8.Folch J., Lees M., Stanley G.H.C. (1957) J. Biol. Chem. **226**, 497.

9.Bragdon J.H. (1951) Biol. Chem. **190**, 513.

10.Stewart C.T., Hendry E.G. (1935) J. Biochem, **29**, 1688.

11.Lowry O.H., Rosebrough N.J., Farr A.L., Randall R.J. (1951) J. Biol. Chem. **193**, 263.

12.Havel R.L., Eder H.A., Bragdon J.M. (1955) J. Clin. Invest. **34**, 1345.

13.Mookerjea E.S., Parks C.E., Kuksis A. (1975) Lipids **10**, 374.

14.Goodridge A.G.(1973) J. Biol. Chem. **248**, 4318.

15.Hoffman M., Weiss L., and Wieland O.H. (1978) Anal. Biochem. **84**, 441.

16.Shefer S., Hauser S., Mosbach E.H.(1978) J. Lipid Res. **9**, 328.

17.Haven G.T., Krzemian J.R., Nguyen T.T. (1973) Res. Commun. Chem. Path. Pharmacol. **6**, 253.

18.Wada F., Hirata K., Sakameto Y. (1989) J. Biochem. **65**, 171.

19.Balasubramaniam S. , Mitropoulos K.A., Venkatesam S. (1978) European J. Biochem. **90**, 377.

20.Greenspan M.D., Lowenstein J.M. (1968) J. Biol. Chem. **243**, 6273.

21.Lamb R.G., Wyrick S.D., Piantadosi C. (1977) Athersclerosis **27**, 147.

22.Mavis R.D., Jacob N., Finkelstein J.N., Hall B.P. (1978) J. Lipid Res. **19**, 467.

23.Chait A., Iverius P.H., Brunzell J.D. (1982) J.Clin. Invest. **69**, 490.

24.Miettinen T.A., Huttunen J.K., Strandberg T., Naukkarinen V., Mattila S., Kumlin T. (1981) Lancet **2**, 478.

**Received: June 24, 1996 - Accepted: July 8, 1996 -**
**Received in revised camera-ready format: July 16, 1996**

